# Social Emotional Learning Program Boosts Early Social and Behavioral Skills in Low-Income Urban Children

**DOI:** 10.3389/fpsyg.2020.561196

**Published:** 2020-11-04

**Authors:** Brian Calhoun, Jason Williams, Mark Greenberg, Celene Domitrovich, Michael A. Russell, Diana H. Fishbein

**Affiliations:** ^1^Human Development and Family Studies, The Pennsylvania State University, University Park, PA, United States; ^2^Substance Use Prevention and Evaluation Research Program, RTI International, Durham, NC, United States; ^3^Edna Bennett Pierce Prevention Research Center, The Pennsylvania State University, University Park, PA, United States; ^4^Department of Psychiatry, Georgetown University, Washington, DC, United States; ^5^Biobehavioral Health, The Pennsylvania State University, University Park, PA, United States; ^6^FPG Child Development Institute, University of North Carolina, Chapel Hill, NC, United States

**Keywords:** social emotional learning, low income, children, social and behavioral skills, physiology, cognition, preventive intervention, moderation

## Abstract

Social emotional learning (SEL) programs are increasingly being implemented in elementary schools to facilitate development of social competencies, decision-making skills, empathy, and emotion regulation and, in effect, prevent poor outcomes such as school failure, conduct problems, and eventual substance abuse. SEL programs are designed to foster these abilities in children with a wide range of behavioral, social, and learning needs in the classroom, including children who are economically disadvantaged. In a previous study of kindergartners residing in a high-poverty community (*N* = 327 at baseline), we observed significant behavioral improvements in children receiving an SEL program—The PATHS^®^ curriculum (PATHS)—relative to an active control condition within one school year. The present investigation sought to determine whether these improvements were sustained over the course of two school years with intervention and an additional year when intervention was no longer provided. Further, using multilevel models, we examined whether baseline measures of neurocognition and stress physiology—known to be adversely impacted by poverty—moderated heterogeneous outcomes. Finally, a preliminary linear regression analysis explored whether neurocognition and physiological stress reactivity (heart rate variability, HRV) predict change in outcomes postintervention. Results confirmed that students who received PATHS sustained significant behavioral improvements over time. These effects occurred for the full sample, irrespective of putative baseline moderators, suggesting that children in high-risk environments may benefit from SEL interventions irrespective of baseline cognitive functioning as a function of overall substantial need. Of interest is that our exploratory analysis of change from waves three to four after the intervention concluded brought to light possible moderation by baseline physiology. Should subsequent studies confirm this finding, one plausible explanation may be that, when an intervention providing protective effects is withdrawn, children with higher HRV may not be able to regulate physiological stress responses to environmental challenges, leading to an uptick in maladaptive behaviors. In reverse, children with lower HRV—generally associated with poorer emotion regulation—may incur relatively greater gains in behavioral improvement due to lesser sensitivity to the environment, enabling them to continue to accrue benefits. Results are discussed in the context of possible pathways that may be relevant to understanding the special needs of children reared in very low-income, high-stress neighborhoods.

## Introduction

Children being raised in underresourced and historically marginalizedcommunities are at a greater risk for behavioral, mental, andacademic problems, largely due to the lack of resources and high rates of exposure to adverse experiences, including chronic poverty, maltreatment, community violence, and structural racism (Clarkson Freeman, 2014; Jones et al., 2016). Chronic and severe adversities of these sorts can exert negative impacts on the circuitry of the brain and perturb the stress response system in ways that increase children’s vulnerability to behavioral and psychological disorders (McEwen, 2008; Loman and Gunnar, 2010; National Scientific Council on the Developing Child, 2014). Further, children living in disadvantaged communities are more likely to exhibit developmental delays in executive functions (EFs) (Kishiyama et al., 2009; Noble and Farah, 2013), such as working memory, inhibiting prepotent responses to extraneous information, and engaging in appropriate goal-directed sustaining and switching of attention (Center for the Developing Child at Harvard University, 2011). These skills are essential for self-regulation and other social emotional competencies, which develop naturally throughout childhood, but are susceptible to impairments in the context of adversity (Blair and Raver, 2016).

Given the negative effects of poverty and associated adversities, preventive interventions have often focused on the low-income children. Programs likely to be most effective address this confluence of factors in a comprehensive fashion by building skill sets, increasing resilience to adversity, and mitigating environments in which children spend a significant portion of their time (Shepard and Dickstein, 2009). Schools constitute an ideal environment in which to implement preventive and promotive interventions given their reach and cost effectiveness (Greenberg et al., 2017). Increasingly, universal school-based interventions that promote social emotional learning (SEL) are being implemented to support the social, emotional, and academic functioning of students and to facilitate the development of competencies that foster mental and behavioral health over time (Durlak et al., 2011; Taylor et al., 2017). SEL programming is most often deployed in early elementary school, a critical period when academic engagement and social–emotional skills set the stage for long-term success (Bierman et al., 2016; Dusenbury and Weissberg, 2017). When delivered with fidelity, SEL programs are considered among the most effective ways to improve outcomes for children across multiple domains of functioning (Greenberg et al., 2017).

Given the links between social–emotional deficits and poor academic performance, SEL programs are important preventive strategies that provide students with supplemental instruction in various social–emotional skill domains and for improving the quality of instruction and climate of classrooms in schools in under resourced communities (Bierman et al., 2016). SEL programs have the potential to also promote resilience for students exposed to adversities improving self-regulation and social competency skills that, in effect, reduce a range of behavioral and peer problems (Domitrovich et al., 2017). Despite the positive effects of school-based SEL programs, the overall effects of these programs are modest, and there is growing demand from the research community for researchers to go beyond questions regarding program effectiveness and to answer questions regarding for whom interventions are most effective (Shonkoff and Fisher, 2013).

In general, students who are lower functioning at the start of an intervention are expected to incur the greatest relative benefits given that they have more room to improve (Greenberg and Abenavoli, 2017). There are likely a number of individual and environmental characteristics that either facilitate or impede SEL program impacts; however, efforts to identify “functional moderators” rather than simply background variables, are scarce. Applying both conceptual and empirical deductions can aid in identifying, *a priori*, the factors that may predict heterogeneity in SEL outcomes. The literature points, in particular, to specific dimensions of EF that are theoretically targeted by SEL program components. One study of the Research-Based Developmentally Informed (REDI) program, a comprehensive preschool intervention that includes the Preschool PATHS curriculum and intervention components to promote children’s early language and literacy skills, explored whether EFs moderated the effects of REDI on child outcomes (Bierman et al., 2008). The study examined seven school readiness outcomes that were targets of the intervention at posttest (end of the Head Start year). Baseline EF abilities, as measured by cognitive tasks (backward word span, peg tapping, and dimensional card sort), did not moderate social or academic outcomes. However, baseline behavioral measures (walk a line slowly and task orientation rated during testing) moderated outcomes; children with lower levels of EF at baseline responded more positively to REDI (Bierman et al., 2008). In a study of the PATHS to PAX intervention, a program for elementary age students that combines the PATHS Curriculum with the Good Behavior Game, stronger effects after 1 year of programming were found for students who began the school year at a lower level of social, emotional, and behavioral functioning, according to teacher ratings (Ialongo et al., 2019).

Executive function development is highly susceptible to adverse environmental conditions and stress (Bremner et al., 2000; Critchley et al., 2000; Mizoguchi et al., 2000; Spear, 2002; Robinson and Kolb, 2004). Studies have shown that children exposed to adversities, such as poverty, develop patterns of behavioral problems that parallel altered neurodevelopment (Glaser, 2000; National Research Council and Institute of Medicine, 2009; Yoshikawa et al., 2012; Cybele Raver et al., 2013) and exhibit related deficits in emergent affective self-regulatory systems (Raver, 2004; Heckman, 2007). In effect, such exposures are associated with deficits and delays in these experience-dependent brain circuits (McCrory et al., 2010) that underlie self-regulatory skills, leading to risk for academic and social failure (Ramey and Ramey, 2004; Diamond and Lee, 2011) and psychopathology (Stanis and Andersen, 2014; Raymond et al., 2018). Given the integral role of exposure to adversity in this developmental cascade, it is also critical to evaluate physiological stress reactivity (as measured in autonomic responses), a dimension of emotion regulation that is equally as influential as EF. In fact, cognitive and affective processes appear to be reciprocal in that effortful cognitive inhibition may be a prerequisite for the ability to self-regulate emotional responses, and at the same time, regulation of affective responses supports the ability to generate effective strategic planning and coping behaviors (Eisenberg et al., 2018). Limited inhibitory cognitive control over emotional arousal has been specifically implicated in aberrant autonomic responses to social and emotional inputs (Beauregard et al., 2001; Quirk and Beer, 2006; Sinha, 2008). As a result, dysregulated behavior may be subserved by individual differences in the cognitive control and affective processing systems that underlie self-regulation.

Following from this body of evidence, it is plausible that both prior cognitive and affective arousal regulatory deficits may affect heterogeneity of response to SEL programming, perhaps especially in very low-income children who commonly experience socioenvironmental risks and often do not have the opportunities and supports for normative skill development and stress modulation (Blair and Raver, 2015). In the current study, we examine the hypothesis that baseline delays or deficits in these regulatory processes may interfere with the program effects given that a certain level of functioning may be prerequisite to assimilating and executing new skills. In addition, although speculative at present, when the programming is withdrawn, impoverished children may experience a setback in any gains made due to ongoing exposure to adversity. The significance of determining whether baseline regulatory functioning predicts differential responses to intervention is in the potential for more targeted programming to improve their development; i.e., aberrant EF and stress physiology will provide curriculum developers with data for optimizing programs and compelling public health and educational policies to further scale SEL strategies.

### Effects of an SEL Curriculum on Self-Regulation

The PATHS^®^ curriculum is a universal SEL program designed to improve skills in four domains: self-control/emotion regulation, attention, communication, and problem solving. Normative improvements in these competencies across development portends healthy behavioral and mental health outcomes. The PATHS curriculum is structured such that training in social competency skills through teacher instruction compensates for deficits and delays, instilling the skills needed to refrain from problem behavior. PATHS is thought to improve outcomes by enabling children to control their behavior in the service of goals, which becomes slowly developmentally coupled with their cognitive and linguistic abilities through the integrated process of linking language, EFs (inhibitory control and planning), and interpersonal interactions (Kusché and Greenberg, 2012). This integrated process of SEL supports both prosocial and positive behavior and recruits newly developed executive and linguistic functions to exert effortful control over behavior in emotional contexts (i.e., frustration, anger).

These processes of social–cognitive maturation are important in achieving socially competent action and healthy peer relations (Do et al., 2019). Of particular importance are the concepts of vertical control and verbal processing of action. Vertical control is the process of higher-order cognitive processes exerting control over lower-level limbic impulses vis-à-vis the development of frontal cognitive control (Luria, 1966). PATHS is designed to consciously teach children skills that reinforce vertical control by providing opportunities to practice conscious strategies for self-control and problem-solving. Acquisition of this skill set builds resilience and is especially critical for children who experience high levels of adversity.

Consistent with expectations, PATHS has been shown to be effective in improving the social and emotion knowledge and self-regulatory skills of children in preschool (Domitrovich et al., 2007) and in Grades 1 through 4 (Greenberg, 2004; Riggs et al., 2006; Panayiotou et al., 2019). However, as expected for a universal intervention, outcomes are heterogeneous, and effect sizes have been relatively modest (0.2 to 0.4). To examine direct effects more closely, our previous paper evaluated the curriculum’s effects on children in kindergarten in urban schools characterized by a high level of poverty and crime (Fishbein et al., 2016). We determined that PATHS conferred beneficial impacts in a single school year, with highly significant effects on the entire array of outcomes. These findings suggested that an SEL-based program, such as PATHS, has potential to alter functioning over a relatively short period of time. Importantly, effect sizes were of considerable magnitude for some outcomes (Cohen *d* of about 0.50 or greater), in contrast with previous studies that found fewer children were benefitting despite statistical significance. Higher effect sizes in this study may be attributed to the intensive coaching that was provided—a prerequisite to identify moderated effects of baseline EF abilities.

### The Current Study

The current investigation examined the effects of PATHS when implemented over a 2-year period (throughout kindergarten and first grade) and includes a follow-up into second grade when the intervention was not delivered. The goal is to determine whether this SEL program has pervasive and sustained positive effects on behavioral, relational, and cognitive abilities in early school-aged children. We also hypothesized that neurocognitive and physiological factors, assessed before and after kindergarten, at the end of first grade, and half way into second grade, would moderate program effects to further elucidate factors that predict heterogeneous outcomes (Schonert-Reichl et al., 2015). In addition, we conducted preliminary analyses to explore whether neurocognitive and physiological processes at baseline predicted change when the intervention was no longer being delivered. The premise behind this analysis is that children who sustain behavioral improvements may be distinguishable from children whose self-regulation declines in the absence of intervention. Such a scenario may be particularly applicable to children living in poverty. Without continued scaffolding from an SEL intervention like PATHS, ongoing exposure to adversity may once again degrade vertical control, allowing behavioral issues to resurface.

## Materials and Methods

### Design Overview

The design for this investigation allowed for a small number of schools to be randomly assigned either the PATHS intervention or a control condition to focus on individual-level differences in direct effects and moderation of those effects. Our intention was not to conduct an effectiveness trial, given that PATHS has been extensively tested and deemed to meet criteria for designation as an evidence-based program^1^. As such, PATHS was an ideal choice for a controlled experiment to determine for whom the intervention works best. We initially identified a number of schools based on kindergarten class size, percentage of students receiving free and reduced lunch (top 1%), mean level of third-grade academic proficiency (bottom 15%), and rates of neighborhood juvenile arrests (averaged about 50% of juveniles between 10 and 17 years old). From this pool, four public elementary schools in Baltimore City were recruited from highly disadvantaged neighborhoods where school readiness is relatively low, and the rates of trauma, drug addiction, and violent crime are high^2^. After obtaining principal and teacher agreement, the schools were randomly assigned to an experimental (PATHS) or control condition (teacher professional development workshops). PATHS is administered grade-wise within a school. The similarity between the communities that the schools serve in terms of sociodemographic mix, crime rates, income level, free or reduced lunch participation, disciplinary rates, and standard achievement scores provides confidence that the student bodies are comparable, and there is little variability in demographic characteristics in these neighborhoods and between the study conditions (see Fishbein et al., 2016).

### Participants

**FIGURE 1:**
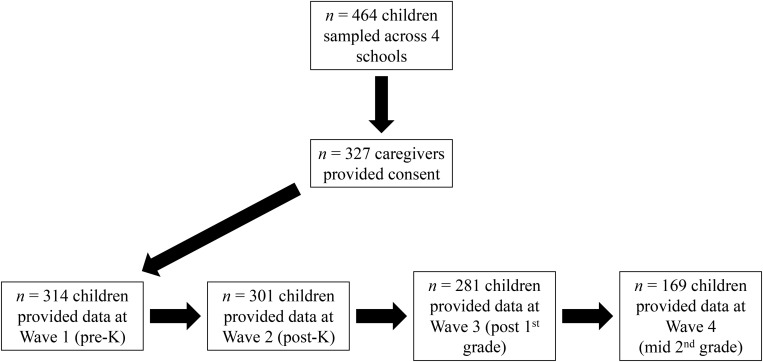
Flow chart of participant recruitment and retention.

Children in the kindergarten classrooms of all four schools were recruited during two staggered waves (in two cohorts) to achieve an adequate sample size per condition (see Fishbein et al., 2016; for details of recruitment). There were approximately 464 children in the four schools, and 327 of them provided caregiver consent based on a combination of the return of signed consent forms and our ability to make direct contact. Of the 327 children whose parents provided caregiver consent, 281 remained in the study through the three waves assessed here (i.e., baseline, post-kindergarten, and post-first grade), and 169 remained through all four waves of data collection (i.e., through mid-second grade) (see Figure 1). Many caregivers were not contactable, did not accompany their children to school, and did not attend school meetings. This scenario is common in high-poverty urban communities, making it difficult to determine reasons for nonresponse or orchestrate a tertiary recruitment strategy.

### PATHS Intervention

The preschool/kindergarten version of the PATHS curriculum was used as the primary intervention in kindergarten, and detailed manuals are available from the publisher (Domitrovich et al., 2004) and the Grade 1 version in first grade (Kusché et al., 2011). This program is organized around a core set of scripted lessons that were taught by teachers twice a week for approximately 20 min and utilized direct instruction, puppet presentations, and stories to help children learn cognitive/behavioral strategies for calming down (e.g., the Turtle Technique), labeling emotions (e.g., Feeling Faces), and problem solving (e.g., The Control Signal). Discussion and role-playing activities provided children with a chance to practice skills and for teachers to monitor students’ level of understanding and skill. Approximately 40% of the lessons focus on skills related to understanding and communicating emotions, 30% focus on skills designed to increase positive social behavior (e.g., social participation, prosocial behavior, and communication skills), and 30% on teaching management and problem solving. Teachers were paid a minimal amount to attend a two-day training each October and January in the delivery of the curriculum by a certified PATHS trainer. They also received ongoing support from a coach who visited the classroom weekly to observe and provide feedback. The coach monitored fidelity and dosage by collecting lesson logs from teachers and conducting classroom observations of program delivery (Fishbein et al., 2016).

### Control Intervention

An active placebo condition in the comparison schools was introduced. This attentional control involved the same incentives for teachers and the school as in the experimental condition, as well as fully supported teacher participation in two 2-h Professional Development Workshops conducted by Dr. Wendy Reinke (co-author of a book entitled Coaching Classroom Management [2008]) who is an expert in teacher consultation and behavioral analysis. The sessions, implemented at the same time as the intervention training (October and January), focused on supporting teachers in managing classroom behavior and maximizing the learning environment. These workshops did not interfere with our case/control comparison as the focus is on behavior rather than socio-emotional development. Also, it was not conducted with sufficient intensity to alter outcomes in the control school. The PATHS trainer observed students/classrooms in the control schools at the same intervals to simulate conditions of the experimental school. Testing of students in the control and intervention schools were equivalent and simultaneous.

### Measures^3^

#### Demographics

We attempted to obtain background information about the child’s home and family life, as well as medical and behavioral history via an initial contact via either telephone, in-person, or mail interview with the primary caregiver. As many caregivers consented to their child’s participation but were not available for this interview, there was a substantial amount of missing background data. Although variability exists in any given population, the primary indicators that would have been measured by our surveys and relevant to our models would have produced fairly uniform information, e.g., with respect to household income, caregiver education, single-parent homes, crime rates, and race/ethnicity.

#### Procedures for Teacher Ratings and Child Testing

We administered all instruments in the beginning of the fall kindergarten semester and during two subsequent spring semesters (kindergarten and first grade) for both students and teachers. As such, students were exposed to PATHS for two school years (K and grade 1). The test battery was administered again halfway through second grade—approximately 7 months postintervention. Children were individually assessed by highly trained master’s level research associates (RAs) who were blinded to condition. There were two test sessions of less than 45 min at each data collection wave.

#### Teacher-Rated Behavioral Measures

Teachers completed a series of measures assessing child competencies. From the *Social Competence Scale* (Conduct Problems Prevention Research Group [CPPRG], 1995), the following subscales were administered: Social Competence (α = 0.87), Prosocial Behavior (α = 0.96), and Emotion Regulation (α = 0.88). *The Teacher Observation of Child Adaptation—Revised* (TOCA—R) (Werthamer-Larsson et al., 1991) assessed overt aggression and internalizing behaviors. To assess Diagnostic and Statistical Manual symptoms of attention-deficit/hyperactivity disorder, teachers completed the *Child Activity Scale* (CAS, known as the ADHD Rating Scale) (DuPaul, 1991) that includes 14 items, segmented into three subscales reflective of inattention-hyperactivity α = 0.92), impulsivity-hyperactivity (α = 0.94), and total score.

Teachers also completed *the Student–Teacher Relationship Scale* (STRS) (Pianta, 2001), which assessed student–teacher closeness (α = 0.90) and conflict (α = 0.92). To assess the quality of peer relations, teachers completed the *Peer Relations Questionnaire* (PRQ), which assesses the degree to which a student was liked and disliked by classmates, left out or ignored, and teased or picked on (α = 0.79) (Ladd and Profilet, 1996). Teachers provided ratings of students’ academic skills by completing four items drawn from the *Academic Competence Evaluation Scales* (α = 0.95) (DiPerna and Elliott, 1999).

#### Cognitive Functioning

##### Intelligence

We used the *KBIT-2*, an estimated intelligence measure that produces two verbal and one nonverbal subscales as well as an intelligence composite score (Kaufman and Kaufman, 1990). The KBIT-2’s internal reliability coefficients for the IQ composite ranges from 0.89 to 0.96 across age groups with slightly lower coefficients for the nonverbal (0.91) and verbal (0.88) subscales; however, nonverbal scale coefficients were as low as 0.78 for children between 4 and 5 years old (Kaufman and Kaufman, 1990).

##### Motor Impulsivity

The *Peg-Tapping Task* assesses working memory and inhibitory control (Diamond and Taylor, 1996). During this task, we instructed participants to tap their peg twice with a wooden dowel when the RA taps once and once when the RA taps twice. Successful task performance requires holding the tapping rule in working memory while inhibiting opposing responses (Pellicano, 2007). After practice trials, participants are administered a series of 16 trials in a pseudorandom sequence (eight one-tap and eight two-tap trials).

##### Delay of Gratification

*Delay of Gratification* (DoG) tasks gauge the ability to delay receipt of an initial smaller reward to attain a larger or more coveted but later reward. Participants were told that they could have a preselected prize contained in a box (i.e., the DoG box) or that they could select any prize from a larger selection box if they could remain seated and refrain from touching the DoG box for 10 min while the experimenter completed paperwork. Key variables generated from this task include “delay” (time waited for reward), “activity level” (rating of degree to which child fidgeted), and “overall difficulty” (rating of difficulty on the part of the child during the waiting period).

##### Behavioral Inhibition

The *Whack-A-Mole* (WAM) is a go/no-go task designed to assess inhibitory control in children. This computerized task presents images in rapid succession of a mole (which occurs more often) or an eggplant popping up in a garden. We instructed participants to press the spacebar on the keyboard whenever the mole appeared but to withhold their response when the eggplant appeared. Shorter reaction times in go trials and higher percentages of correct responses (i.e., fewer commission errors) in no-go trials are associated with greater inhibition and emotion regulation (Hirose et al., 2012).

#### Psychophysiology Protocol

Autonomic physiology was recorded at intervention baseline prior to and during completion of the *MacArthur Story Stem Battery* (MSSB) (Bretherton and Oppenheim, 2003), which guides children to represent social relationships in situations of conflict. We used the task to induce a mild level of stress for measurement of physiological reactivity using three story stems. The first was neutral/positive prompt (birthday party) and was followed by two challenging social scenarios (one depicting social isolation and one depicting social conflict). The RA provided the child with small figurines for each character in the story as well as any relevant props associated with the story stem. The RA began each story stem following a standardized script and used the figurines and props to play-act the story. The child was then prompted to continue the scene, using the figurines, to complete the story. Each story stem was ended when the child reached what the RA perceived to be the “peak” of activity. At that point, they were asked “how do you feel about what’s happening in the story?” If the story appeared to reach a plateau with no further change or impending resolution, the RA would ask “how does the story end/stop/finish?” Total duration of story time was a mean of 4.55 min (*SD* = 1.92).

Cardiac data were recorded from 3 Ag/AgCl disposable spot electrodes placed on the child’s torso. Resting physiological activity was recorded for 3 min prior to the start of the story stem task and throughout the task. Physiological data were extracted across the two stressor stories to ensure sufficient recording time. Because children differed in their self-generated responses to the story stems, length of response time for each stem varied. For responses that exceeded 3 min, RAs manually selected a 3-min window in consultation with the video recording to ensure that the selected 3 min were best matched to the affective content of the response.

##### Heart Rate Variability (HRV)

Data were collected continuously at 500 Hz and a bandpass filter of 0.5 and 45 Hz, via a MindWare Technologies ambulatory recording unit that transmitted wirelessly to a laptop running BioLab software v3.0. Data were processed by Vivonoetics Inc., where staff reviewed raw electrocardiograph data to identify and correct any erroneous or missing beats in the cardiac series. For any portion of data contaminated by noise affecting the identification of more than two consecutive beats, the affected portion of the data series was removed. After cleaning, data were processed in the time domain, root mean square of the successive differences (RMSSD), according to published guidelines (Force, 1996). RMSSD is considered an accurate snapshot of the autonomic nervous system’s parasympathetic branch and was used herein as the basis for our HRV score. Reactivity scores were computed as the rest period preceding the task minus the stressor condition. Positive reactivity scores for HRV indicate parasympathetic withdrawal during the social stress stories (increased arousal).

### Analytic Strategy

Multilevel models, estimated using the PROC MIXED procedure in SAS 9.4 (SAS Institute Inc., Cary, NC, United States), were used to estimate the impact of the PATHS intervention on change in children’s behavioral, social, and academic outcomes. Models included both intercept and slope random effects, which allowed for interindividual variation in children’s baseline level and rate of change in outcomes.

In models testing for direct effects of PATHS from pre- to post-intervention (i.e., Waves 1–3), male sex was grand-mean centered, and waves 1, 2, and 3 were coded as 0, 1, and 2, respectively, so that the intercepts could be interpreted as the average value of the outcome variable for the average child in the control group, rather than for only females in the control group. The group variable was uncentered. The main estimate of the effect of PATHS was the wave × group interaction, β_11_. A Cohen’s *d* statistic was used as a measure of effect size, and it was computed by taking the difference between the slope estimates of the PATHS group and the control group (i.e., β_11_ − β_10_), multiplying this difference by the time interval between pre- and postintervention (i.e., 2 years), and then dividing this product by the standard deviation of the outcome at preintervention, as described by Feingold (2009). This statistic indicated how many standard deviations PATHS changed the growth rate (i.e., slope) for each outcome across the duration of the intervention.

After testing for direct effects, wave 1 neurocognitive and physiological variables were added to the models to test whether they moderated the effects of PATHS. This was done by adding three additional parameters to each growth model: the level-2 neurocognitive/physiological variable, a cross-level wave × neurocognitive/physiological variable interaction, and a cross-level wave × group × neurocognitive/physiological variable interaction. The equation used to test for moderation of PATHS effects on child outcomes was:


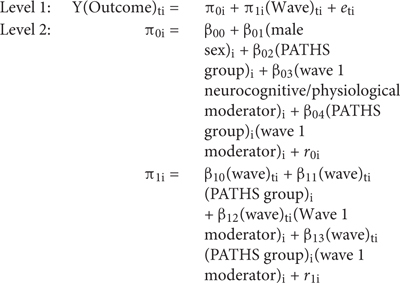


Eight neurocognitive variables measured at preintervention (i.e., wave 1) were tested as moderators of intervention effects including three delay of gratification variables (activity level, delay in minutes, and overall difficulty), three behavioral inhibition variables (mean accuracy on the go and no-go tasks and mean response time on the go task), IQ, and motor impulsivity (peg tapping). Three wave 1 physiological variables related to HRV were also tested as moderators of intervention effects including neutral, positive, and negative RMSSD. All moderator variables were standardized. The three-way wave × group × neurocognitive/physiological variable interaction, β_13_ indicated whether each neurocognitive/physiological variable moderated the effects of PATHS. Cohen’s *d* effect sizes for these tests of moderation were calculated by taking the difference between the coefficient of the three-way wave × group × moderator interaction and the two-way wave × moderator interaction (β_13_ – β_12_), multiplying this difference by the time interval between pre- and postintervention, and dividing this product by the standard deviation of the outcome at baseline (Feingold, 2009).

Finally, exploratory moderation analyses were performed to see if the preintervention neurocognitive and physiological variables predicted differential change in outcomes from postintervention to 6-month follow-up. These analyses used linear regressions to test whether each of the neurocognitive and physiological variables predicted scores on outcomes at follow-up (i.e., wave 4) while controlling for post-intervention scores (i.e., wave 3) of the outcome. These models only included children from the treatment group and used the following equation:

Y(wave 4 outcome)_I_ = B_0_ + B_1_(wave 3 outcome)_i1_ + B_2_(neuro/physio variable)_i2_ + e_i_.

## Results

### Descriptive Statistics

Of the 327 children whose parents provided caregiver consent, 310 children provided data on the outcome variables assessed here in at least one of the first three waves (i.e., pre- to postintervention) and were included in the longitudinal models used to test the first two hypotheses. The analytic data set contained 7.45% missing data, and observations that included complete data were included in analyses (i.e., listwise deletion was used to handle missing data). Baseline univariate descriptive statistics for outcome and neurocognitive/physiological moderator variables are presented in Tables 1 and 2, respectively. Of the 310 children in the analytic sample, 169 (54.52%) were female, and 150 (48.39%) were in the PATHS treatment group.

**TABLE 1:** Descriptive statistics of outcome variables at wave 1 (preintervention).

	PATHS Group				Control Group			
	*N*	*M* (*SD*)	Min.	Max.	*N*	*M* (*SD*)	Min.	Max.
Aggression	149	1.84 (0.87)	1.00	4.86	151	1.80 (1.04)	1.00	5.57
Internalizing	149	2.11 (0.69)	1.00	4.17	151	1.74 (0.75)	1.00	4.33
Total social competence	149	3.68 (0.91)	1.77	6.00	151	4.74 (1.02)	1.54	6.00
Emotion regulation	149	3.89 (0.92)	1.33	6.00	151	4.64 (1.08)	1.50	6.00
Prosocial behavior	149	3.50 (0.97)	1.71	6.00	151	4.83 (1.09)	1.57	6.00
**Child activity scale**								
Impulsivity	149	1.60 (0.69)	1.00	3.75	151	1.67 (0.78)	1.00	4.00
Inattention	149	1.64 (0.62)	1.00	3.83	151	1.68 (0.81)	1.00	4.00
Total	149	1.62 (0.63)	1.00	3.71	151	1.68 (0.75)	1.00	4.00
**Student–Teacher relationship scale**								
Closeness	149	4.06 (0.66)	2.25	5.00	151	4.51 (0.63)	2.12	5.00
Conflict	149	1.69 (0.94)	1.00	4.88	150	1.56 (0.81)	1.00	4.88
Total	149	4.19 (0.69)	2.00	5.00	151	4.48 (0.63)	2.19	5.00
**Peer relationships**								
Questionnaire total	149	1.94 (0.64)	1.00	4.00	151	1.45 (0.69)	1.00	4.00
Academic skills total	149	3.18 (1.19)	1.00	5.00	149	3.58 (1.13)	1.00	5.00

**TABLE 2:** Descriptive statistics of neurocognitive and physiological moderator variables at wave 1 (preintervention).

	PATHS Group				Control Group			
	*N*	*M* (*SD*)	Min.	Max.	*N*	*M* (*SD*)	Min.	Max.
**Delay of gratification**								
Activity level	137	1.01 (.75)	0.00	3.00	152	0.92 (0.78)	0.00	3.00
Delay in minutes	137	5.51 (2.63)	0.00	7.00	152	5.51 (2.76)	0.00	7.00
Overall difficulty	137	1.14 (1.77)	0.00	4.00	152	0.97 (1.70)	0.00	4.00
**Inhibition**								
Go accuracy	133	0.93 (.09)	0.49	1.00	157	0.90 (0.12)	0.10	1.00
No-go accuracy	133	0.71 (.21)	0.05	0.98	157	0.72 (0.22)	0.07	1.00
Go response time (s)	133	712.77 (118.78)	479.31	1,097.64	157	735.04 (137.26)	302.43	1,154.12
IQ (composite standard score)	138	89.27 (11.06)	58.00	116.00	154	90.09	53.00	119.00
Peg tapping, total correct	149	12.19 (4.83)	0.00	16.00	159	13.32 (3.62)	1.00	16.00
**Root mean square of the successive differences (RMSSD)**								
Neutral	95	62.13 (38.45)	9.30	186.19	123	69.13 (43.66)	3.03	252.74
Positive	96	60.24 (37.57)	10.42	182.26	122	66.23 (38.76)	3.23	249.09
Negative	96	59.31 (34.10)	12.17	183.32	120	62.09 (35.46)	3.52	194.72

### Hypothesis 1: Direct Effects of PATHS From Pre- to Postintervention

The results of all 13 models testing for direct effects of PATHS on child behavioral, social, and academic outcomes are presented in Table 3. Males reported significantly *worse* preintervention scores than females on all 13 outcomes, β_01_. However, since gender had no significant effect on rates of change over time (analyses not reported here), it was not included as moderator of change over time. Preintervention internalizing, total social competence, emotion regulation, and prosocial behavior scores were *worse*, on average, for children in the PATHS group than for children in the control group, β_02_. For example, the model-estimated preintervention internalizing score was 1.69 for the average child in the control group and 1.99 for the average child in the PATHS group. Similarly, preintervention STRS closeness, STRS total, PRQ total, and academic skills total scores were *worse*, on average, for children in the PATHS group than for children in the control group. In contrast, there were no differences in preintervention scores between the control and PATHS groups for aggression, any of the three CAS outcomes, or STRS conflict.

**TABLE 3:** Direct effects of the PATHS intervention on child behavioral, social, and academic outcomes.

Outcome variable	Initial status			Slope	
	Intercept, β_00_	Male gender, β_01_	PATHS group, β_02_	Wave, β_10_	Wav × Group, β_11_
	β (SE)	β (SE)	β (SE)	β (SE)	β (SE)
Aggression	1.78 (0.07)***	0.40 (0.10)***	−0.03 (0.11)	0.32 (0.05)***	−0.22 (0.07)**
Internalizing	1.69 (0.06)***	0.14 (0.07)*	0.30 (0.09)***	0.18 (0.04)***	−0.28 (0.06)***
Total social competence	4.90 (0.08)***	−0.30 (0.10)**	−1.06 (0.11)***	−0.36 (0.06)***	0.85 (0.08)***
Emotion regulation	4.78 (0.08)***	−0.30 (0.10)**	−0.74 (0.11)***	−0.33 (0.06)***	0.66 (0.08)***
Prosocial behavior	5.00 (0.08)***	−0.32 (0.10)**	−1.33 (0.12)***	−0.39 (0.06)***	1.01 (0.09)***
**Child activity scale**					
Impulsivity	1.64 (0.06)***	0.34 (0.07)***	−0.11 (0.08)	0.10 (0.03)**	−0.06 (0.05)
Inattention	1.63 (0.06)***	0.32 (0.08)***	−0.05 (0.08)	0.14 (0.03)***	−0.11 (0.05)*
Total	1.63 (0.05)***	0.33 (0.07)***	−0.08 (0.08)	0.12 (0.03)***	−0.08 (0.04)
**Student–teacher relationship scale**					
Closeness	4.61 (0.05)***	−0.15 (0.06)**	−0.46 (0.07)***	−0.20 (0.04)***	0.43 (0.05)***
Conflict	1.50 (0.07)***	0.29 (0.09)**	0.12 (0.10)	0.27 (0.05)***	−0.13 (0.07)
Total	4.56 (0.05)***	−0.22 (0.07)**	−0.29 (0.07)***	−0.23 (0.04)***	0.28 (0.05)***
**Peer relationships**					
Questionnaire total	1.41 (0.06)***	0.19 (0.07)**	0.45 (0.08)***	0.24 (0.04)***	−0.37 (0.06)***
Academic skills total	3.64 (0.09)***	−0.40 (0.12)***	−0.40 (0.13)**	−0.11 (0.06)*	0.32 (0.08)***

*N* = 795–798 observations nested within 308–310 persons. *p < 0.05; **p < 0.01; ***p < 0.001.

With respect to differences between groups over time (i.e., throughout the 2-year duration of the intervention), children who received the PATHS intervention showed significantly greater improvement than children in the control group, β_11_, in aggression, internalizing, total social competence, emotion regulation, and prosocial behavior. For example, model-estimated aggression scores for the average child in the control group increased from 1.78 at preintervention to 2.42 at postintervention, whereas aggression scores for the average child in the PATHS group increased at a *significantly slower rate* from 1.75 to 1.95 across the duration of the intervention. Emotion regulation scores for the average child in the control group *decreased* from 4.78 at preintervention to 4.12 at postintervention, whereas emotion regulation scores for the average child in the PATHS group *increased* from 4.04 to 4.70 throughout the intervention. The magnitude of the effect of PATHS was large (i.e., *d* > 0.80; Cohen, 1988, 1992) on all five of these outcomes with effect sizes of *d* = −1.13 on aggression, *d* = −1.23 on internalizing, *d* = 2.20 on total social competence, *d* = 1.85 on emotion regulation, and *d* = 2.28 on prosocial behavior.

Children who received the PATHS intervention demonstrated significantly greater improvement than children in the control group, β_11_, on only one of the three CAS outcomes. Model-estimated CAS inattention scores for the average child in the control group children increased from 1.63 at preintervention to 1.91 at postintervention, whereas CAS inattention scores for the average child in the PATHS group increased significantly *less* from 1.58 to 1.64 across the duration of the intervention. The size of the effect of PATHS on change in CAS inattention scores was medium (*d* = −0.69). In contrast, average CAS impulsivity and total scores increased throughout the intervention, but the average rates of change did not differ between children in the control and PATHS groups.

Children who received PATHS showed significantly more improvement, β_11_, on average, than children in the control group on two of the three STRS outcomes. Specifically, model-estimated STRS closeness scores for the average child in the control group *decreased* from 4.61 at preintervention to 4.41 at postintervention, whereas STRS closeness scores for the average child in the PATHS group *increased* from 4.15 to 4.61 across the duration of the intervention. STRS total scores for the average child in the control group children *decreased* from 4.56 at preintervention to 4.10 at postintervention, whereas STRS total scores for the average child in the PATHS group *increased* from 4.27 to 4.37. The sizes of the effect of PATHS on change in STRS closeness (*d* = 1.83) and STRS total (*d* = 1.58) scores were large. In contrast, average STRS conflict scores increased throughout the intervention, but the average rates of change did not differ between children in the control and PATHS groups.

Last, children who received PATHS showed significantly greater improvement than children in the control group, β_11_, on average, in PRQ total and academic skills total scores. Model-estimated PRQ total scores for the average child in the control group children *worsened* from 1.41 at preintervention to 1.89 at postintervention, whereas PRQ total scores for the average child in the PATHS group *improved* from 1.86 to 1.60 throughout the intervention. The size of the effect of PATHS on change in PRQ total scores was large (*d* = −1.72). Teacher-rated academic skills total scores for the average child in the control group decreased from 3.64 at preintervention to 3.42 at postintervention, whereas academic skills total scores for the average child in the PATHS group increased from 3.24 to 3.66 across the duration of the intervention. The size of the effect of PATHS on change in academic skills total scores was medium (*d* = 0.74). Taken together, children who received the PATHS intervention showed significantly greater improvement over time, on average, in 10 of 13 outcomes, and these effects were mostly large (*d* > 0.80).

### Hypothesis 2: Moderation of PATHS Effects

After testing for direct effects, separate models were estimated that added preintervention measurements of neurocognitive and physiological variables to determine whether these variables moderated the effect of PATHS on change in children’s behavioral, social, and academic outcomes. Of the 143 models tested for moderation (11 moderators × 13 outcomes), only five (3.5%) were statistically significant. Peg tapping moderated the effect of PATHS on STRS closeness scores, no-go mean accuracy moderated the effect of PATHS on STRS conflict scores, and all three physiological variables moderated the effect of PATHS on PRQ total scores. Given the overall pattern of findings and that alpha was set at 0.05, these five statistically significant findings were most likely due to chance (i.e., they were Type I errors). Further, the sizes of the moderating effects were mostly negligible (*d* < 0.10 or 0.20). Therefore, our findings failed to support our second hypothesis that neurocognitive and physiological variables would moderate the effects of PATHS on change in children’s behavioral, social, and academic outcomes.

### Hypothesis 3: Exploring Differential Change in Outcomes in the Posttreatment Phase

Linear regressions were conducted to explore whether sustained behavioral improvement after intervention (i.e., from postintervention to 6-month follow-up) could be differentiated on the basis of preintervention neurocognitive and physiological variables. For the neurocognitive potential moderators, only 1 of the 117 models was significant and, thus, could be considered due to chance. For the physiological potential moderators, 6 of the 26 models predicted statistically significant change after the intervention concluded. Specifically, preintervention (i.e., wave 1) neutral RMSSD was *inversely* associated with change in total scores for STRS closeness, STRS total, and academic skills from postintervention (i.e., wave 3) to 6-month follow-up (i.e., wave 4). Preintervention-positive RMSSD was *inversely* associated with change in prosocial behavior and STRS closeness scores in the posttreatment phase. Preintervention-negative RMSSD was *inversely* associated with change in STRS closeness scores in the posttreatment phase. Although the few significant findings were possibly Type I errors, because nearly all the variables predicting change from postintervention to 6-month follow-up were physiological, this may suggest that baseline HRV is involved in differential responsivity to program effects after the conclusion of the intervention.

## Discussion

The present investigation was designed to evaluate the impacts of an SEL intervention—PATHS—on a range of behaviors in young school-aged children residing in high-poverty, urban neighborhoods. Based on a substantial body of research establishing the negative effects of poverty and trauma on neurocognitive functioning and stress physiology (National Scientific Council on the Developing Child, 2014), we were particularly interested in the extent to which baseline differences in these processes predicted intervention outcomes. If level of functioning prior to intervention influences ultimate outcomes, then children in most need might be least likely to benefit from programming. The importance of this line of inquiry is reflected in the premise that interventions could potentially be constructed to more directly target those mechanisms that would otherwise interfere with program impacts on children’s behavior.

However, contrary to our original hypotheses, we did not find significant moderation by neurocognitive or psychophysiological variables on outcomes at the end of 2 years of intervention. Rather, direct effects of PATHS on multiple behavioral outcomes of interest were strong in this population of children who participated in this intervention, while children who received the control treatment exhibited relative declines rather than gains in several outcomes. None of the putative neurocognitive or physiological moderators impacted the growth of skills as a result of the PATHS intervention.

Results showing a wide range of direct effects of this universal intervention in young children suggest that PATHS may truly exert universal benefits. We found improvements in peer and teacher relations, prosocial behavior, internalizing behaviors, social competencies, and academic performance ratings, among others. Rose’s Paradox (Rose, 1981) may provide some insights into the significance of these findings by proffering that reducing overall behavioral problems in young school-aged children may have greater population level effects than focusing only on the highest-risk children, which is where our focus was originally directed (Greenberg and Abenavoli, 2017). Although less serious, most poor behavioral outcomes are not found only within that highest-risk group. Rose would argue that greater societal gain may be obtained by achieving a small reduction in poor behavioral outcomes within a far larger group of “risky” children with less serious problems than by trying to reduce problems among a smaller number of children with very serious problems. Perhaps that scenario applies to the present results.

On the other hand, the social/demographic context studied here may play a role in the ability of PATHS to exert such strong effects and should be considered when formulating interpretations, potentially lessening the relevance of Rose’s Paradox in this case. It is plausible that the broad impacts of PATHS we observed irrespective of “risk” status—as measured in baseline neurocognitive and stress physiological responses—may be specifically applicable to low income, disadvantaged children with a prevalence of trauma, neglect, and food insecurity. The program may be conferring protective effects against ongoing exposure to adversity. As positive outcomes were not exhibited by children in classrooms that received a control condition (i.e., improvements in instructional methods), it is unlikely that just any type of attention to a high-need population—that often lacks basic supports at home—is responsible for the gains and that components of the SEL intervention can be credited with the observed benefits. PATHS focuses on building self-regulatory and social skills that are instrumental in navigating adverse and stressful environments (Kusché and Greenberg, 2012), thereby facilitating adaptive behaviors in the context of less than optimal circumstances. The impacts of such program components are, thus, expected to be widely experienced throughout this population as a result, while preexisting conditions become relatively less potent.

Although intervention effects remained significant, they plateaued over time. Our first article with this cohort, examining short-term change from pre- to post-kindergarten in response to PATHS, reported strong effect sizes for nearly all outcomes (Fishbein et al., 2016). That initial inoculation, during a year when children are entering public school and are developmentally better prepared for greater immersion in social settings, may have conferred the largest boost to behavioral regulatory, social competency, and academic skills, after which benefits appeared to be sustained. After two academic years of intervention exposure, during a period when PATHS was no longer offered, no additional benefits were incurred. We might speculate that consolidation of skills would normalize development over time in this population if the intervention was continuously implemented or if its active ingredients were infused into teaching practices. Our findings also call into question whether, within the intervention group, children who continue to improve after the program ends fundamentally differ from those who show a decay in skill level. Two possible explanations for such differences are that: (1) in the absence of intervention, the ongoing experience of adversity may enable dysregulatory behaviors to resurge (Tolan et al., 2020), or (2) individual level differences in functioning (e.g., stress physiology) at baseline may set these children apart. In the present investigation, we were able to only preliminary explore the latter explanation, as discussed below.

### Exploring Moderation After Intervention Concluded

Although we did not find evidence of moderation by any of our neurocognitive or physiological variables across all measurement occasions, an examination that focused specifically on change from waves 3 to 4 after the intervention concluded brought to light possible moderation by baseline physiology. Of the seven models that showed moderation effects, six included baseline RMSSD (neutral, positive, and negative conditions), suggesting that higher levels of HRV may predict declines in behavioral improvements after receiving PATHS. Stress physiology is arguably a more apt reflection of the degree to which stressful experiences alter bodily systems in any given individual and, thus, constitute more sensitive measures of potentially prognostic factors than tallies of traumatic incidences or surveys of perceived stress. Increased HRV at baseline may indicate a higher susceptibility to environmental influences, translating to an overreaction both physically and behaviorally to high social demands (Dale et al., 2011). As such, when an intervention providing protective effects is withdrawn, children with higher HRV may not be able to regulate physiological stress responses to challenges in their environment, leading to an uptick in maladaptive behaviors. In reverse, children with lower HRV—generally associated with poorer emotion regulation—may have incurred relatively greater gains in behavioral improvement. Their lesser sensitivity to the environment, hypothetically speaking, may enable them to continue to accrue benefits in response to program-taught skills.

An intriguing pathway yet to be explored may be relevant to the premise of the current study and these preliminary findings. HRV, a well-characterized biomarker of stress reactivity, has been consistently associated with the effectiveness of cognitive control over emotion regulation (Holzman and Bridgett, 2017). Chronic and/or severe stress adversely impacts this top–down process, which is marked, in part, by suppression of HRV. Neurobiological substrates of cognitive and emotion regulatory processes, such as the prefrontal cortex (PFC) and amygdala, are coincidentally altered across development in response to stress; HRV and functionality of these structures are highly interrelated (Steinfurth et al., 2018). In particular, the ventro-medial prefrontal cortex (vmPFC), an aspect of the PFC, plays a key role in conditioned fear responses and perceptions of threat via its connection with the amygdala (Motzkin and Koenigs, 2015). Studies have shown less activity and smaller volume in vmPFC in individuals exposed to severe stress (e.g., adults with PTSD) (Motzkin and Koenigs, 2015), including poverty (Javanbakht et al., 2015). Relatedly, adults raised in impoverished environs appear to be more sensitive to social threat cues and less sensitivity to positive social cues, outcomes that are unpinned by PFC and amygdalar activity and connectivity (Javanbakht et al., 2015). As such, it is possible that greater HRV levels at baseline may portend worse outcomes after intervention due to heightened sensitivity to the environment in concert with compromised top–down neurobiological control as a function of poverty and trauma. In addition, children who exhibit lower HRV levels at baseline may be less sensitive to adverse conditions, counterbalancing adversity-induced “damage” to these brain structures, enabling them to continue to benefit from newly learned skills. Although aspects of these relationships have been charted, the full pathway has yet to be explored. Regardless, these very preliminary findings require confirmation before this interpretation can be considered.

Inclusion of biologically based moderators of behavioral change in response to psychosocial preventive interventions for behavioral problems are exceedingly rare (Fishbein et al., 2006), and those that do, largely examine pre–post intervention effects with few exceptions (Glenn et al., 2018); most do not include follow-up measurement occasions. In fact, such modeling also typifies intervention studies in other fields, including medicine, psychology, and psychiatry. Findings, to date, in the field of prevention have been unimpressive for the most part, showing only modest influences from biological moderators on outcomes that, in turn, decay over time (Nigg et al., 1999). Fishbein and colleagues (2006) found that the level of neurocognitive functioning at baseline was significantly predictive of response to a violence preventive intervention in at-risk minority adolescents from high-poverty neighborhoods in a microtrial. Two additional such investigations of the Head Start REDI program in socially and economically disadvantaged children reported that dimensions of EF moderated program impacts on school readiness (Bierman et al., 2008; Sasser et al., 2017). Neither study included a distal follow-up measurement. A few studies that evaluated mindfulness-based programs have not shown significant impacts on clinical or behavioral outcomes when measured pre- and postintervention; however, in their evaluation of follow-up indicators months after intervention concluded, improvements in measured outcomes surfaced (Fjorback et al., 2013; Butzer et al., 2017). Further examination suggested that recipients who continued to incorporate the practices into their behavioral repertoire after the intervention ended constituted a subgroup evincing the greatest benefits, while others showed diminishing returns. Such findings raise the possibility that participants who consolidate behavioral change after the intervention ends are distinctive from those who simply receive intervention and revert to behavior-as-usual. To discern the differences, at least one additional measurement occasion distal to program conclusion is needed, along with pertinent moderators that may help explain these distinctive pathways. Furthermore, including physiological monitoring in preventive intervention studies holds potential to reveal underlying mechanisms in differential responsivity. Given the likely heterogeneity in this group, future studies to identify distinctive clusters based on physiology and receptivity to intervention would provide further direction in determining best practices for vulnerable populations.

### Limitations

One of the limitations of this study was that we were unable to assess parent-reported baseline risk status on an individual basis given the relative inaccessibility of their caregivers. Such measurements would have been highly advantageous to determine whether individual-level stress exposures, and other contextual and experiential data at baseline may have moderated program impacts. A second limitation was the use of behavior ratings rather than direct observations of child behavior. Although there are problems inherent in teacher ratings, particularly when they are collected from teachers who also deliver the intervention, the fact that an intervention effect was found on ratings by three different teachers (Kindergarten, Grade 1, and Grade 2) lends credibility to the findings. The second-grade teacher ratings after the intervention had ended add to our confidence. Regardless, future research would benefit from verification of teacher-reported effects with behavioral observations. Another limitation was that analyses were conducted at the individual child level even though the unit of randomization was the classroom. As this was primarily a study of mechanisms that required neurocognitive and physiological testing, the small number of classrooms did not provide sufficient statistical power to use multilevel models. The clustering of students within classrooms results in the non-independence of subjects, an assumption inherent in the analyses conducted in this study. It is possible that this could bias the statistical tests used to identify intervention effects.

## Conclusion

Our findings were not supportive of original hypotheses that neurocognition and emotion regulation would predict intervention responsivity; expectations were that children with lower levels of functioning would not benefit from PATHS to the extent that higher functioning children would. Instead, all children benefitted significantly irrespective of baseline functioning. We have surmised that direct effects were “universal” due to the high level of need in this population. The children included in this study were very low income or under the poverty level, and trauma in the form of child maltreatment, neglect, witnessing violence, caregiver addiction, and many other adverse childhood experiences are commonplace in these Baltimore neighborhoods. In essence, these children may have been primed for absorbing a nurturing, SEL program provided by schools. Benefits plateaued to some extent after the intervention ended, suggesting that positive effects may not be sustained over time without ongoing SEL programming or boosters in this population. In other words, prevailing adverse experiences in the absence of programming may diminish gains made when actively in intervention. Results of this study should compel both policy changes that reduce childhood exposure to trauma, as well as educational investments in child health and well-being by providing ongoing programming in high-need communities.

## Data Availability Statement

The raw data supporting the conclusions of this article will be made available by the authors, without undue reservation.

## Ethics Statement

The studies involving human participants were reviewed and approved by Institutional Review Board, RTI International. Written informed consent to participate in this study was provided by the participants’ legal guardian/next of kin.

## Author Contributions

BC ran the data analyses and wrote the statistical and results sections. DF conceived of the project and wrote the introduction, methods, and discussion. DF and MG designed the study. CD led the intervention arm. JW was involved in the project throughout the entire period of performance, processing the data, and performing early data analyses. MR consulted on the statistical technique. All authors provided input and edited the manuscript.

## Conflict of Interest

The authors declare that the research was conducted in the absence of any commercial or financial relationships that could be construed as a potential conflict of interest.

## Footnotes

1 https://www.preventionresearch.org/advocacy/#SofE

2 https://health.baltimorecity.gov/state-health-baltimore-winter-2016/state-health-baltimore-white-paper-2017

3 Measures are more fully described with respect to scale items, scoring algorithms, and alphas in Fishbein et al. (2016).
